# Engineering Additive Manufacturing and Molding Techniques to Create Lifelike Willis’ Circle Simulators with Aneurysms for Training Neurosurgeons

**DOI:** 10.3390/polym12122901

**Published:** 2020-12-03

**Authors:** Pin-Chuan Chen, Jang-Chun Lin, Chung-Hsuan Chiang, Yi-Chin Chen, Jia-En Chen, Wei-Hsiu Liu

**Affiliations:** 1 Department of Mechanical Engineering, National Taiwan University of Science and Technology, Taipei 106, Taiwan; Pcchen@mail.ntust.edu.tw (P.-C.C.); M10703117@mail.ntust.edu.tw (C.-H.C.); giokgiok0711@gmail.com (Y.-C.C.); 2 High Speed 3D Printing Research Center, National Taiwan University of Science and Technology, Taipei 106, Taiwan; 3 Department of Radiation Oncology, Shuang Ho Hospital, Taipei Medical University, Taipei 110, Taiwan; 13451@s.tmu.edu.tw; 4 Department of Radiology, School of Medicine, College of Medicine, Taipei Medical University, Taipei 110, Taiwan; 5 Medical 3D Printing Center, Tri-Service General Hospital and National Defense Medical Center, Taipei 114, Taiwan; w112233442@gmail.com; 6 Department of Biomedical Engineering, Tri-Service General Hospital and National Defense Medical Center, Taipei 114, Taiwan; 7 Department of Neurological Surgery, Tri-Service General Hospital and National Defense Medical Center, Taipei 114, Taiwan; 8 Department of Surgery, School of Medicine, National Defense Medical Center, Taipei 114, Taiwan

**Keywords:** neurosurgeon surgical simulator, aneurysm clipping surgery practice, fully transparent and elastic vascular Simulator, additive manufacturing, molding, and dissolution

## Abstract

Neurosurgeons require considerable expertise and practical experience in dealing with the critical situations commonly encountered during difficult surgeries; however, neurosurgical trainees seldom have the opportunity to develop these skills in the operating room. Therefore, physical simulators are used to give trainees the experience they require. In this study, we created a physical simulator to assist in training neurosurgeons in aneurysm clipping and the handling of emergency situations during surgery. Our combination of additive manufacturing with molding technology, elastic material casting, and ultrasonication-assisted dissolution made it possible to create a simulator that realistically mimics the brain stem, soft brain lobes, cerebral arteries, and a hollow transparent Circle of Willis, in which the thickness of vascular walls can be controlled and aneurysms can be fabricated in locations where they are likely to appear. The proposed fabrication process also made it possible to limit the error in overall vascular wall thickness to just 2–5%, while achieving a Young’s Modulus closely matching the characteristics of blood vessels (~5%). One neurosurgical trainee reported that the physical simulator helped to elucidate the overall process of aneurysm clipping and provided a realistic impression of the tactile feelings involved in this delicate operation. The trainee also experienced shock and dismay at the appearance of leakage, which could not immediately be arrested using the clip. Overall, these results demonstrate the efficacy of the proposed physical simulator in preparing trainees for the rigors involved in performing highly delicate neurological surgical operations.

## 1. Introduction

Neurosurgeons treating cerebral aneurysms require a thorough understanding of the position of the aneurysm relative to the parent artery and its branches, the surrounding brain regions, and neurocranium. They also require highly developed surgical skills, which necessitates extensive practice [1]. However, as hospital administrators seek to improve patient safety by reducing the workload of junior neurosurgeons, it is becoming increasingly difficult for trainees to find opportunities for practical training by reducing the workload of junior neurosurgeons and limiting access to all but the most highly trained [1,2]. The use of physical simulation as a proxy for practical experience is gaining wide support, as indicated by the following statement issued by the Association of American Medical Colleges in 2011: “Simulation has the potential to revolutionize healthcare and address patient safety issues if appropriated utilized and integrated into the educational and organizational improvement process” [3,4,5].

At present, simulation tools used for the training of neurosurgeons have two types, including computer-based [6,7,8,9,10,11] or physical devices manufactured with rapid prototyping techniques [12,13,14,15,16,17,18,19]. The use of virtual reality (VR) technology for the training of surgeons was first introduced by Lanier in 1987. In the same period, Delp et al. [6] reported on the first VR simulation for the repair of the Achilles’ tendon, and numerous computer-based simulations have been developed since. Pelargos et al. [9] presented a comprehensive review of VR and augmented reality (AR) in the training of neurosurgeons, including the history, potential, current status, and limitations. VR and AR can be used to learn patient-specific anatomy, plan responses to complications, select appropriate surgical instruments, and enhance operational efficiency. Enabling neurosurgeons to learn and rehearse surgical procedures eliminates the risk to patients and reduces the likelihood of surgical error. Choudhury et al. [10] was the first to use a VR simulator to standardize training for neurosurgical oncology. Experiment results demonstrated the efficacy of this approach for the preparation of neurosurgical residents and the development of technical skills. Gmeiner et al. [11] developed a patient-specific virtual simulation of aneurysm clipping with haptic force feedback and real-time deformation of the vessel and aneurysm wall.

Despite the considerable advances in VR and AR technologies that have been reported [20], computer-based simulation tools were less beneficial to the surgical skill development, primarily due to the lack of tactile feedback [8]. D’urso et al. [12] and Wurm et al. [13] designed the first cerebrovascular simulators, in which stereolithography (SL) was used to create aneurysms and the surrounding blood vessels. They demonstrated the efficacy of physical simulators in providing an overview of relevant anatomic structures, planning surgical procedures, and selecting appropriate aneurysm clips. Advances in rapid prototyping, such as 3D printing, have greatly expanded the availability of simulators for research and training. Ripley et al. [14] used SL in conjunction with 3D printing to fabricate simulators of the aortic root complex for use in training surgeons to perform transcatheter aortic valve replacement (TAVR) in specific patients. They demonstrated the feasibility of using 3D printing to create physical simulators as a noninvasive technique to assist in the 3D visualization of patient-specific aortic root anatomy. Torres et al. [15] used a 3D printer to create a simulation system for endovascular aneurysm repair (EVAR), which was shown to improve the performance of surgical residents by reducing fluoroscopy time by 30% and procedure time by 29%. Wurm et al. [16] used SL in conjunction with 3D printing to create a solid aneurysm with the surrounding vessels and neurocranium to provide practice in performing clipping surgeries. They described 3D printing as the most promising rapid prototyping technique for creating neurovascular structures. Kimura et al. [17] used 3D printing to create a hollow 3D biomodel of semi-elastic aneurysms on which to perform craniotomy, requiring drilling of the skull base to gain access to aneurysms and clipping. They also fabricated an aneurysm with surrounding vessels and cranial base bones mimicking difficult clinical cases. Mashihiro et al. [18] employed 3D printing to create a solid aneurysm using acrylonitrile butadiene styrene (ABS), which was then coated with pink liquid silicone. The subsequent use of xylene to dissolve the ABS left a hollow 3D device with the elastic properties required to simulate the clipping of aneurysms. Liu et al. [19] used fused deposition modeling (FDM) to create a hollow model of the skull, brain, and aneurysm, which was then coated with silicone and dissolved to leave a hollow device with realistic elasticity. Benet et al. [21] reported on using a 3D printer to create a patient-specific aneurysm for implantation in a human cadaver to facilitate training in complex case management and intricate surgical procedures. Experiment results demonstrated the efficacy of that approach in exposing surgical trainees to realistic situations requiring clipping within specific surgical corridors.

Cadavers can be used to give trainees practical experience navigating the structure of the brain under the trained eye of senior surgeons; however, cadavers are expensive and difficult to obtain, and the vascular morphology of cadavers often differs from that of patients. Despite recent advances in VR technology, such systems remain expensive, the imaging modalities differ from those encountered in real-world 3D spaces, and they lack tactile feedback. Most existing 3D-printed medical simulators lack dimensional accuracy, realistic elasticity, and the transparency required to provide meaningful insights into the structures relevant to surgical operations.

In the current study, we integrated molding technology with additive manufacturing and ultrasonic dissolution to create highly detailed models of complex anatomic structures, including hollow transparent blood vessels with realistic dimensions and flexibility. The Circle of Willis was used to demonstrate the proposed fabrication scheme, due to its complex structure connecting multiple major arteries in the brain and its importance when accessing aneurysms. The resulting model of the brain stem, soft brain tissue, carotid arteries, and a Circle of Willis was evaluated in terms of dimensional accuracy and material properties. Neurosurgical trainees were then given the opportunity to gain hands-on experience performing surgical procedures, including the clipping of aneurysms under the guidance of senior neurosurgeons. The proposed simulator proved highly effective in evaluating the quality of clipping procedures and providing experience in dealing with cases of aneurysm rupture during surgery.

## 2. Fabrication of Simulators

The medical simulation device in the current study included the skull, brain stem, soft brain tissue, elastic carotid arteries, and a fully transparent Circle of Willis vascular system. Note that fabricating and fixing the Circle of Willis vascular system was challenging, due to its complex structure and location deep within the brain. There are several additive manufacturing processes involved to create a lifelike and whole simulator, and reviews of various additive manufacturing techniques are widely reported [22].

### 2.1. Circle of Willis Vascular System

**Figure 1:**
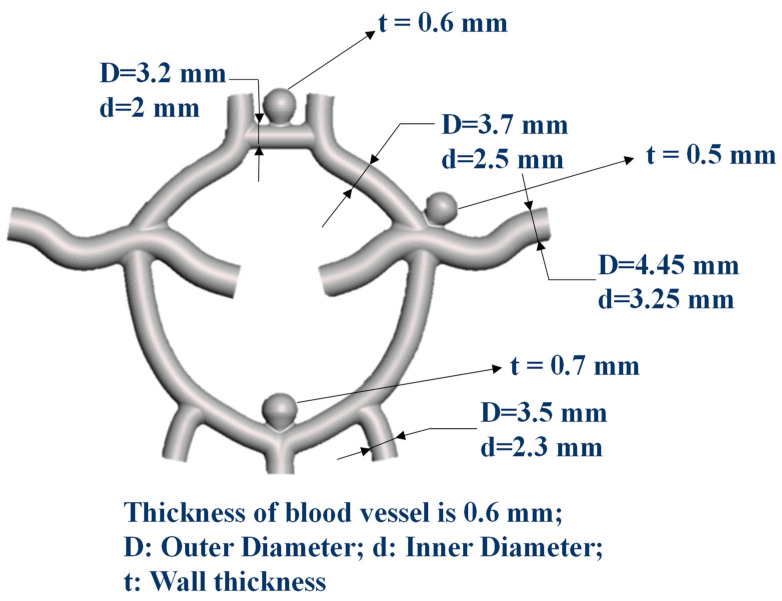
A layout of the Circle of Willis vascular system, showing the obvious differences in the outer diameter (D), inner diameter (d), and wall thickness (t) in various locations.

**Figure 2:**
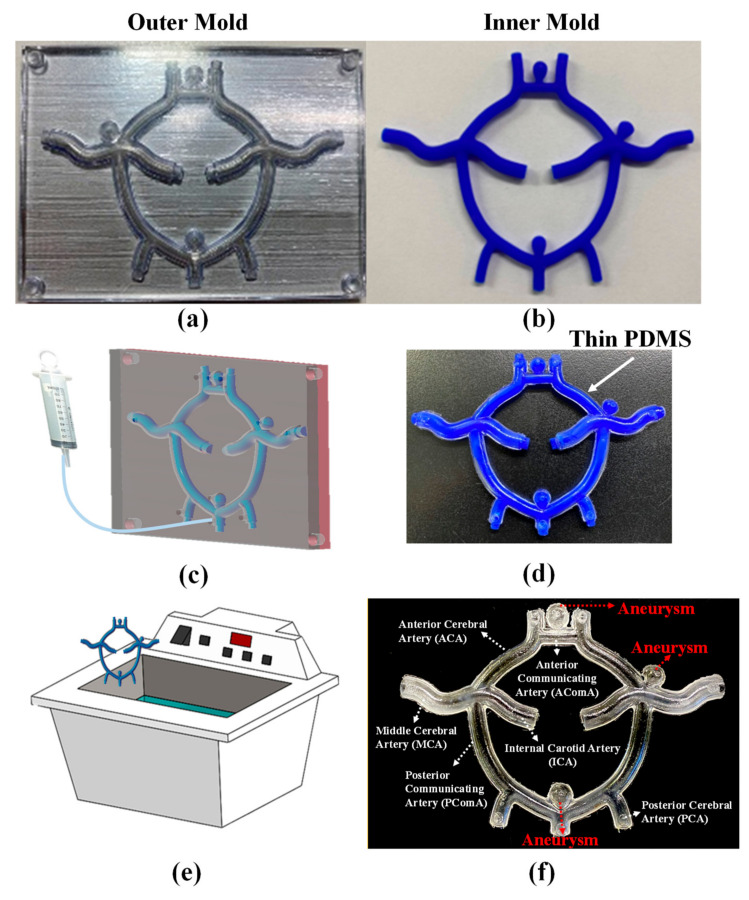
(**a**) The two outer (clamshell) molds fabricated using a commercial 3D printer (Objet30 Dental Prime, Stratasys) with a biocompatible material (MED 610, Stratasys); (**b**) the inner wax mold fabricated using a commercial 3D printer (Projet 3500 CPX Max, 3D Systems) with a wax material (Visijet Hi-Cast, 3D Systems); (**c**) PDMS was slowly injected into the gap between the molds until the gap was completely filled; (**d**) the PDMS casting (with inner mold still intact) following removal of the outer molds; (**e**) dissolution of the inner wax mold was facilitated using ultrasonication, as reported previously [23]; (**f**) the resulting Circle of Willis with major corresponding arteries.

Figure 1 presents a layout of the Circle of Willis vascular system, showing the obvious differences in the outer diameter (D), inner diameter (d), and wall thickness (t) in various locations. Three aneurysms were designed and located in the three regions of the Circle of Willis vascular system where they are most likely to appear. As shown in Figure 2, fabrication of the Circle of Willis vascular system involved creating three 3D-printed molds, casting elastic materials within the mold assembly, and the ultrasonication-assisted dissolving of the mold materials.

Figure 2a presents the two outer (clamshell) molds fabricated using a commercial 3D printer (Objet30 Dental Prime, Stratasys, Eden Prairie, Min, USA) with a biocompatible material (MED 610, Stratasys, Min, USA). Figure 2b presents the inner wax mold fabricated using a commercial 3D printer (Projet 3500 CPX Max, 3D Systems, Rock Hill, SC, USA) with a wax material (Visijet Hi-Cast, 3D Systems, Rock Hill, SC, USA). The printed products from these two machines have low surface roughness and high dimensional accuracy, which would lead to a fully transparent Circle of Willis with precise dimension. Note that the alignment between the outer and inner molds defined the thickness and uniformity of the vascular walls. Alignment was ensured by carefully designing connectors between the outer and inner molds. Polydimethylsiloxane (PDMS) was slowly injected into the gap between the molds until the gap was completely filled (Figure 2c). Note that several vents were included in the mold design to allow the evacuation of air during the injection of PDMS mixture. Figure 2d shows the PDMS casting (with inner mold still intact) following removal of the outer molds. Dissolution of the inner wax mold was facilitated using ultrasonication, as reported previously [23]. The primary objective of ultrasonication was to induce cavitation within the liquid phase. Cavitation refers to the formation and growth of gaseous microbubbles, the subsequent collapse of which generates microjets and microconvection currents [24]. The effects of ultrasonication penetrated the PDMS to facilitate the transport of solvent into the narrow channels, thereby accelerating the removal of wax. Figure 2f shows the resulting Circle of Willis with major corresponding arteries.

### 2.2. Brain Lobes and Skull

**Figure 3:**
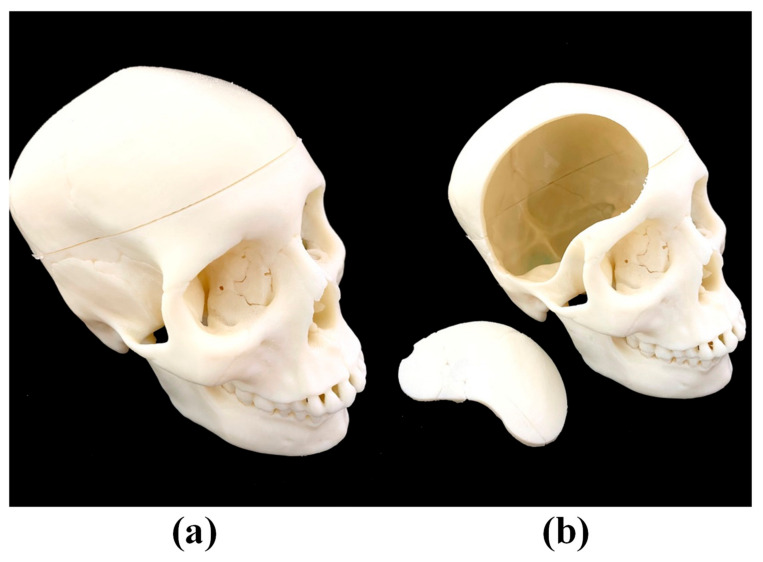
(**a**) The overall 3D-printed skull; (**b**) how a piece of cranium located on the right side of the forehead can be removed to simulate a craniotomy.

3D printing and casting were used again to fabricate the lobe of the brain and surrounding skull. A digital file of the brain lobes and skull was obtained by scanning physical medical models used at Tri-Service General Hospital via an industrial 3D scanner (Artec Space Spider, Artec3D, Luxembourg). An FDM 3D printer (Fortus 360mc, Stratasys, Eden Prairie, Min, USA) was then used to print the skull with acrylonitrile butadiene styrene (ABS) at a relatively lower price. Figure 3a shows the overall 3D-printed skull, and Figure 3b shows how a piece of cranium located on the right side of the forehead can be removed to simulate a craniotomy.

**Figure 4:**
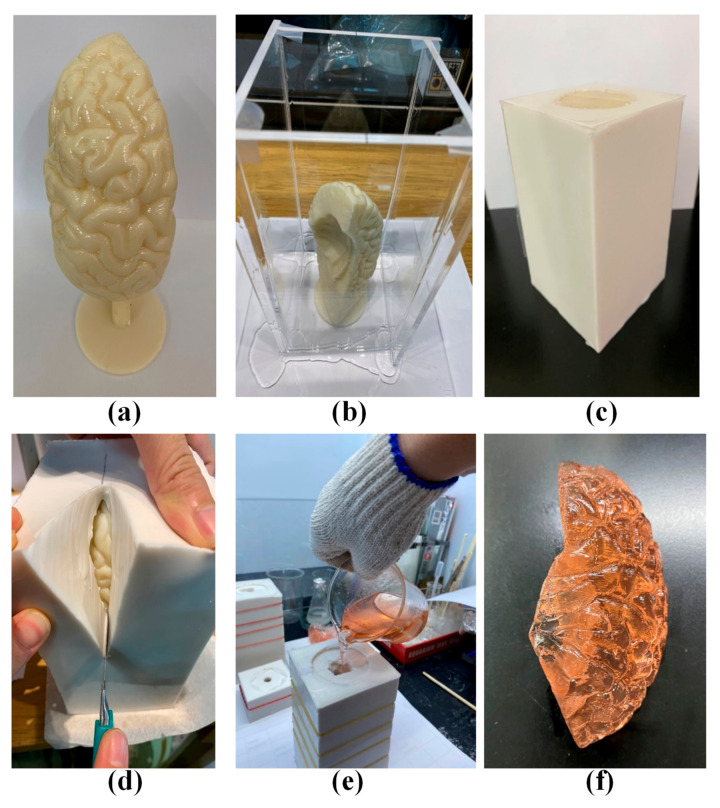
(**a**) A mold of the left frontal lobe printed using a 3D printer (Fortus 360mc, Stratasys); (**b**,**c**) the acrylonitrile butadiene styrene (ABS) mold was fixed within a transparent box into which silicon was poured to create a cast; (**d**) the ABS mold was sliced open using a knife and then peeled back to remove the ABS mold; (**e**) a mixture of pink jelly was poured into the cavity; (**f**) a realistic model of the frontal lobe was created.

The brain lobes were fabricated in four pieces: Frontal lobe, parietal lobe, temporal lobe, and occipital lobe. In the following, the left frontal lobe is used to demonstrate the fabrication process, which included 3D printing a mold followed by multiple castings. Figure 4a shows a mold of the left frontal lobe printed using a 3D printer (Fortus 360mc, Stratasys, Eden Prairie, Min, USA). As shown in Figure 4b, the ABS mold was fixed within a transparent box into which silicon was poured to create a cast (Figure 4c). The ABS mold was sliced open using a knife and then peeled back to remove the ABS mold (Figure 4d). This left a cavity into which a mixture of pink jelly was poured (Figure 4e) to create a realistic model of the frontal lobe (Figure 4f). The other lobes were created using the same fabrication process, as shown in the Supplementary Materials (Figure S1).

### 2.3. Assembly of Complete Simulation Device

**Figure 5:**
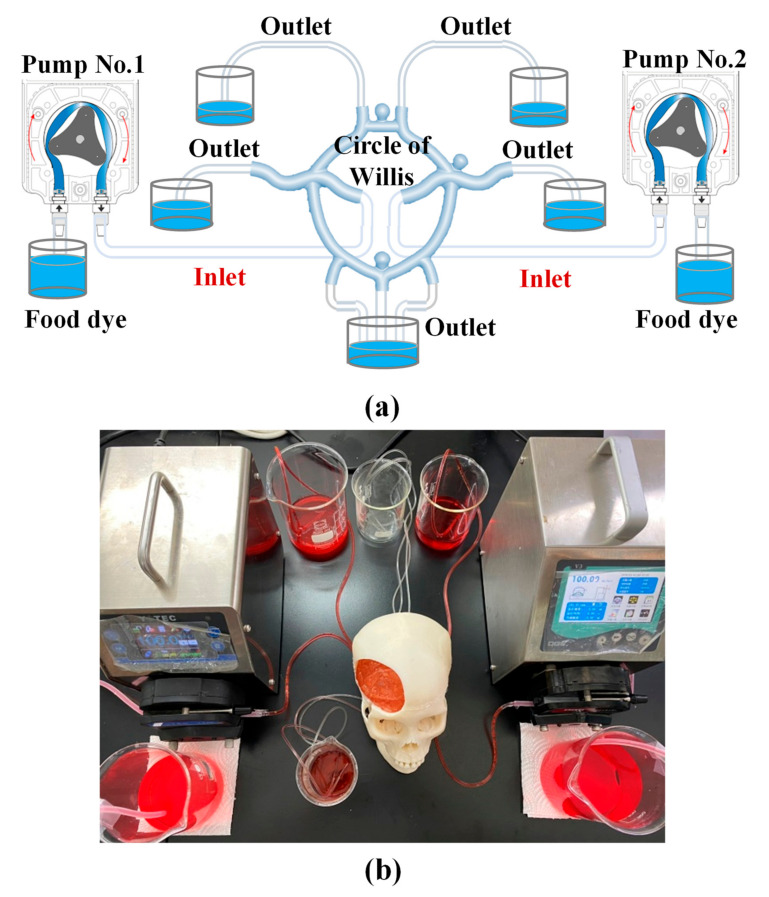
(**a**) The circulatory system, which included two pumps and several beakers; (**b**) the overall system specifically for practicing the clipping of aneurysms during surgery.

Following assembly of the components (Circle of Willis, four brain lobes, and skull), the completed simulation devices was connected to a pumping system to simulate the flow of blood through the brain. Figure 5a presents the circulatory system, which included two pumps and several beakers. Figure 5b presents the overall system, which was set-up specifically to practice the clipping of aneurysms during surgery. During the surgery practice, a food dye solution was pumped into the circulatory system at a flow rate of 146 mL/min [25], whereupon it flowed into the Circle of Willis via two inlets (representing the internal carotid artery (ICA)) and then drained from the assembly via multiple outlets (representing other arteries).

## 3. Experiment Methods

### 3.1. Materials of Circle of Willis

Tactile feedback is the most important aspect of physical simulators, and their primary advantage over digital simulation methods. The casting material in this study was PDMS, an elastomeric material, which enables high-fidelity casting, excellent transparency, and variable elasticity. PDMS mixtures comprise a base elastomer and a curing agent, the ratio of which can be varied to adjust the elasticity. Note that elasticity is the primary factor providing tactile feedback during the clipping of aneurysms. Thus, we produced PDMS mixtures at ratios of 10:1, 12.5:1, 15:1, and 20:1, and analyzed the results using a standard tension test (ASTM D412-16 Standard Test Methods for Vulcanized Rubber and Thermoplastic Elastomers Tension). Note that five specimens of each ratio were fabricated and tested. We compared the Young’s Modulus estimated from tension tests with that of blood vessels [26] in order to determine the ideal base-to-curing agent ratio for our Circle of Willis model.

### 3.2. Visualization of Transparent Vascular System

**Figure 6:**
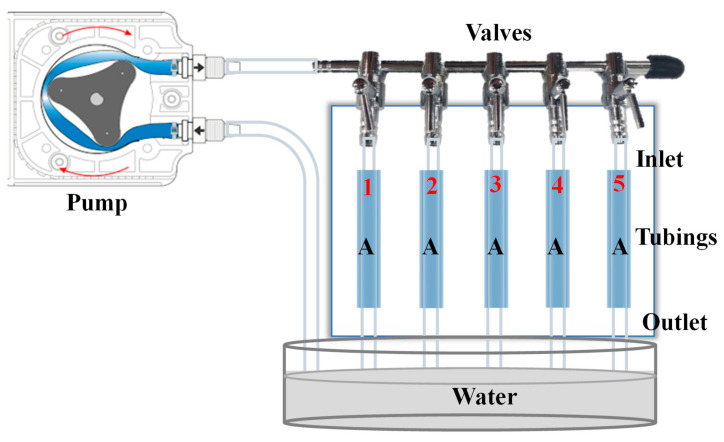
The five tubes were connected to the pumping system with pieces of papers marked with the letter “A” placed beneath. This experiment clearly illustrates the differences in transparency among the five tubes.

Transparency is another important factor determining the effectiveness of surgical training models. Neurosurgical trainees must be able to observe the flow of blood while clipping the aneurysm, if they are to make informed surgical decisions. We evaluated the transparency of five hollow tubes with identical dimensions (inner diameter = 4 mm, outer diameter = 6 mm, and outer length = 40 mm) fabricated using five different methods. The five fabrication methods were as follows: (1) Tube 1 was created using the methods described previously in this paper; (2) Tube 2 was created using a fused deposition modeling (FDM) printer followed by solvent evaporation polishing [26]; (3) Tube 3 was also created using an FDM printer, but without solvent evaporation polishing [26]; (4) Tube 4 was created using a commercial 3D printer (Form 3, formlabs, Somerville, Massachusetts, United States); (5) Tube 5 was created using a different commercial 3D printer (J750/J735, Stratasys, Eden Prairie, Min, USA). As shown in Figure 6, the five tubes were connected to the pumping system with pieces of papers marked with the letter “A” placed beneath. This experiment clearly illustrates the differences in transparency among the five tubes.

### 3.3. Dimensional Accuracy of Vascular System

Tactile feeling of the Circle of Willis is key to this lifelike simulator, and the tactile feeling came from the material and the dimension of the vascular system. The material has been considered in Section 3.1; therefore, the dimensional accuracy of the vascular system will be considered in this section. As shown in Figure 2f, we estimated the dimensional accuracy of the proposed Circle of Willis simulator by comparing measurements of the cross-sectional area of the Circle of Willis in the following locations: AComA, PComA, MCA, PCA, and at aneurysms. At each location, four samples were obtained for measurement using a tool microscope (Leica optical Microscope, DM series). The average error was then calculated as follows:

(1) $$\mathrm{Error}=\;\frac{\mathrm{Measured}\;\mathrm{Value}-\mathrm{Designed}\;\mathrm{Value}}{\mathrm{Designed}\;\mathrm{Value}}\;\times 100\%$$

### 3.4. Training Neurosurgeons in Clipping Aneurysms

Neurosurgical trainees require repeated practice under the guidance of a senior doctor on angling the head, opening the skull, opening the soft tissues of the brain using forceps, and locating the aneurysm. As shown in Figure 5b, pre-operative training exercises for aneurysm clipping were conducted under the supervision of a senior neurosurgeon at the National Defense Medical Center for neurosurgical trainees. In this exercise, trainees also learned to deal with blood leakage during the operation.

## 4. Experiment Results

### 4.1. Using Silicone for the Circle of Willis

**Figure 7:**
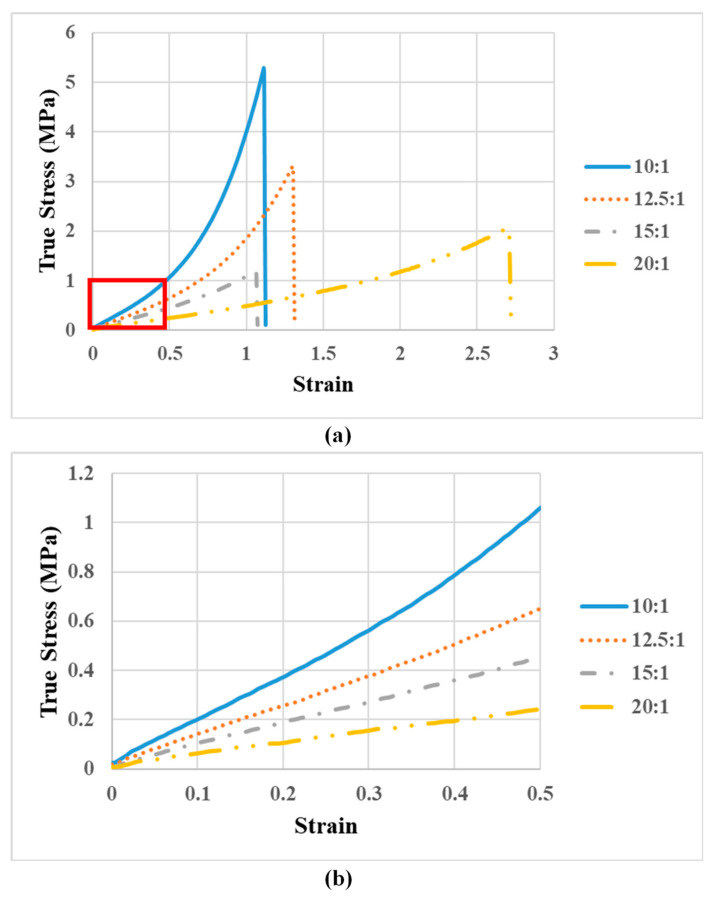
(**a**) The tensile test results of specimens fabricated using different ratios of silicone base and hardener (10:1 to 20:1); (**b**) enlarged area of (**a**), and the Young’s Modulus corresponding to different ratios was estimated from the slopes of each curve.

Figure 7a presents the tensile test results of specimens fabricated using different ratios of silicone base and hardener (10:1 to 20:1). Figure 7b is the enlarged area of Figure 7a, and the Young’s Modulus corresponding to different ratios is estimated from the slopes of each curve; the tensile measurements are detailed in Figure S2. The Young’s Modulus values were as follows: 10:1 (2.06±0.17 MPa), 12.5:1 (1.23±0.09 MPa), 15:1 (0.94±0.02 MPa), and 20:1 (0.47±0.04 MPa). Steiger [26] reported that the Young’s Modulus of cerebral blood vessels is roughly 2.5±1.1 MPa; therefore, we adopted a PDMS mixture ratio of 10:1 for the Circle of Willis.

### 4.2. Transparency of Simulated Blood Vessels

**Figure 8:**
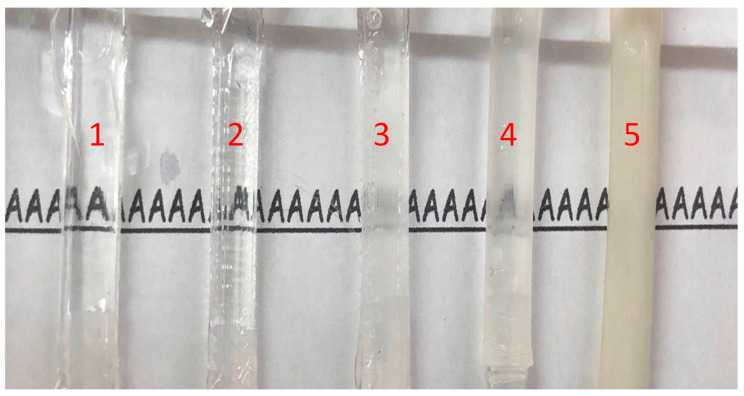
The transparency of the various tubes used to simulate blood vessels. Obviously, the letter “A” appears more clearly through Tube 1, which was fabricated using the method proposed in Section 3.2.

Figure 8 presents the transparency of the various tubes used to simulate blood vessels. Obviously, the letter “A” appears more clearly through Tube 1, which was fabricated using the method proposed in Section 3.2. Overall, it appears that the surface roughness of the mold played a crucial role in determining the transparency of the final tube. To quantify the transparency of those tubings, a spectrophotometer will be used in the near future [27].

### 4.3. Dimensional Accuracy

Table 1 lists the dimensions of blood vessels measured in various locations, including the AComA, MCA, PComA, and PCA, as shown in Figure 2f. The designed thickness of the blood vessels and aneurysms is shown in Figure 1. Based on Equation (1), the average dimensional error in the thickness of blood vessels was estimated at 2.25–5.5%, whereas the standard deviation in the dimensions of the simulator as a whole was 2–4%.

### 4.4. Pre-Operative Training

**Figure 9:**
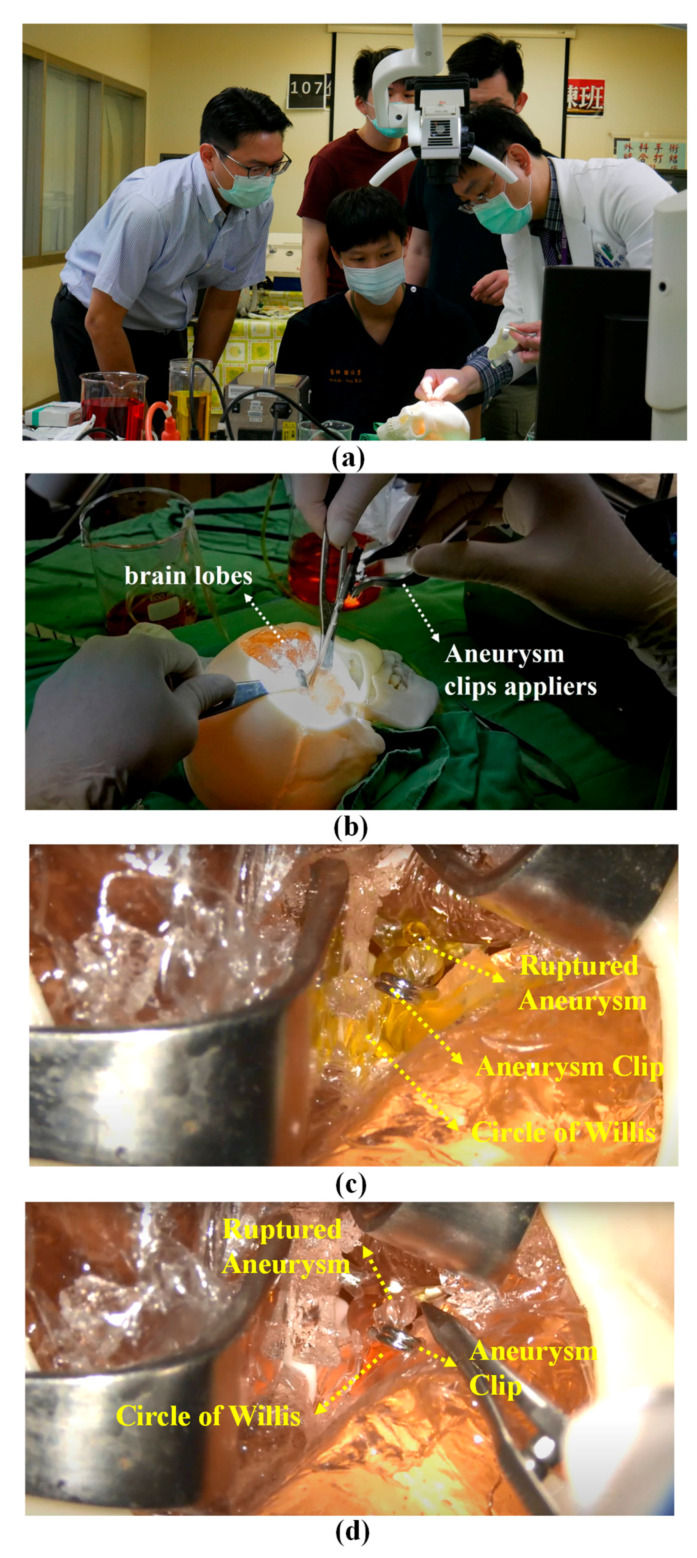
(**a**) An experienced neurosurgeon (Dr. Liu from our research team) gave a neurosurgical trainee instructions on the use of the proposed simulator to practice clipping aneurysms; (**b**) the overall procedure involved a craniotomy followed by the use of surgical forceps to make space between the brain lobes in order to locate the aneurysm; (**c**) based on observations of yellow liquid leaking from the area of interest, the trainee determined that the aneurysm had ruptured. Despite initial efforts to clip the aneurysm, the leakage continued; (**d**) efforts to re-clip the aneurysm under the guidance of Dr. Liu succeeded in stopping the leakage.

As shown in Figure 9a, an experienced neurosurgeon (Dr. Liu from our research team), gave a neurosurgical trainee instructions on the use of the proposed simulator to practice clipping aneurysms. Note that the instructional procedures are detailed in the supplementary video. The aneurysm in the practice session in Figure 2f is located in the ACA, which required adjustment of the head position to a specific angle before surgery commenced. The overall procedure involved a craniotomy followed by the use of surgical forceps to make space between the brain lobes in order to locate the aneurysm (Figure 9b). Throughout the procedure, the flow of blood through the vessels allowed participants to assess the stability and tightness of the clip and determine the success of the procedure. Based on observations of yellow liquid leaking from the area of interest, the trainee determined that the aneurysm had ruptured. Despite initial efforts to clip the aneurysm, the leakage continued (Figure 9c). Efforts to re-clip the aneurysm under the guidance of Dr. Liu succeeded in stopping the leakage. Red liquid dye was then pumped into the circulation system to determine the extent to which the flow of liquid from the artery into the aneurysm had been staunched (Figure 9d). No leakage or mixing of red and yellow dyes was observed by either participant. The trainee therefore withdrew the forceps and covered the skull to complete the operation.

In a subsequent interview, the trainee reported that the physical simulator gave him a deeper understanding of the overall operation, as well as experience of the tactile sensations involved in clipping an aneurysm. The trainee described his strong emotional reaction when he realized that he was unable to halt the leaking. It appears that the proposed physical model performed as intended. That is, this device can effectively assist trainees in learning to deal with situations one could expect to encounter in a real-world surgical environment.

## 5. Conclusions

The training of neurosurgeons requires professional knowledge as well as practical experience in performing difficult operations. The conventional approach to training using cadavers is expensive and does not lend itself to repeated practice sessions. It is also a poor simulation of real-world operating scenarios. Our objective in the current study was to create a lifelike physical simulation device to enable trainees to practice clipping aneurysms. The proposed simulator includes the brain stem, soft brain tissue, carotid arteries, and a hollow transparent Circle of Willis. The fabrication process, combining additive manufacturing with molding techniques, casting, and ultrasonication-assisted dissolution, resulted in a lifelike Circle of Willis with accurate dimensions, tactile feedback, and transparency to enable the checking of blood flow. The assembled device is ideally suited to learning the surgical procedure and dealing with situations one could expect to encounter in a real-world surgical environment.

## Figures and Tables

**Table 1:** The measured error and standard deviation (STD) of arteries manufactured by the proposed fabrication process in this study.

Locations	Average Error (%)	Average Standard Deviation (STD) (%)
AcomA	2.75	3.5
MCA	5.5	3.5
PcomA	2.75	2.75
PCA	5	2
Aneurysm	2.25	4

## Supplementary Materials

The following are available online at https://www.mdpi.com/2073-4360/12/12/2901/s1, Figure S1: (a) parietal lobe; (b) occipital lobe; (c) frontal lobe; (d) frontal lobe, Figure S2: (a) 5 tensile test results with 10:1 PDMS mixture; (b) enlarged area in (a) marked in red rectangle; (c) 5 tensile test results with 12.5:1 PDMS mixture; (d) enlarged area in (a) marked in red rectangle; (e) 5 tensile test results with 15:1 PDMS mixture; (f) enlarged area in (a) marked in red rectangle; (g) 5 tensile test results with 20:1 PDMS mixture; (h) enlarged area in (a) marked in red rectangle. Video S1: demonstration of using lifelike simulator to train neurosurgical trainee.
